# Recurrent thrombocytopenia and Lyme disease

**DOI:** 10.1186/1471-2334-13-S1-P81

**Published:** 2013-12-16

**Authors:** Egidia Miftode, Dănuț Teodor, Roxana Lungu, Cristina Petrovici

**Affiliations:** 1 “Gr.T.Popa” University of Medicine and Pharmacy, Iaşi, Romania; 2 Hospital of Infectious Diseases, Iaşi, Romania

## Background

Mild or moderate thrombocytopenia is a common feature in Lyme disease, but the recurrence of such a severe form of thrombocytopenia is very rare, and the improving knowledge of atypical possibility of evolution could help clinicians to establish an early diagnosis and treatment by the inclusion of specific serology in the protocol of evaluation.

## Case report

We present the case of a 24 year-old man admitted to the Hospital of Infectious Diseases for headache, myalgia, arthralgia and a previous unrepeated episode of moderate fever. Physical examination at the admission revealed a poor general status, loss of appetite, difficulties in maintaining orthostatic position due to the hypotonia of the lower limbs muscles, absence of meningeal irritation syndrome. He denied any tick bites in the past. Lumbar puncture, cranio-cerebral computed tomography were normal, as well as different serology for EBV, *Mycoplasma*, HIV, syphilis. The most prominent hematologic feature was a severe thrombocytopenia (29,000/cmm).

He received ampicillin and dexamethasone and the level of platelets increased progressively to normal during a period of eight days. He was discharged without any complaints, but he returned after 5 days with the same symptoms, and thrombocytopenia (37,000/cmm). A serology test for *Borrelia* was performed and it was positive revealing the diagnosis of Lyme disease. The complete remission of thrombocytopenia under the same association of drugs was very rapid – in 4 days. He was discharged after having been treated for three weeks with antibiotic and two weeks of corticotherapy.

